# Fundamental Motor Skills of Children in Deprived Areas of England: A Focus on Age, Gender and Ethnicity

**DOI:** 10.3390/children5080110

**Published:** 2018-08-13

**Authors:** Leanne Jaye Adeyemi-Walker, Michael Duncan, Jason Tallis, Emma Eyre

**Affiliations:** Centre for Sport, Exercise and Life Sciences, Coventry University, Coventry CV1 2DS, UK; aa8396@coventry.ac.uk (M.D.); ab0289@coventry.ac.uk (J.T.); ab2223@coventry.ac.uk (E.E.)

**Keywords:** fundamental motor skills, motor development, childhood, ethnicity

## Abstract

This study compared the mastery of fundamental motor skills (FMS) of males and females in early-childhood (four to five years, *n* = 170) and in middle-childhood (nine to ten years, *n* = 109) who attend schools in deprived and ethnically diverse areas of England. Process FMS (object control and locomotor skills) were observed using the Test of Gross Motor Development-2. Sprint speed over 10 meters and jump distance assessments were conducted using light gates and tape measures. A gender (male vs. female) by year-group (early-childhood vs. middle-childhood) interaction was shown for the process and product-oriented FMS measurements. Middle-childhood males and females demonstrated significantly greater FMS mastery, as compared to early-childhood (*p* < 0.05). Furthermore, middle-childhood males demonstrated significantly greater mastery of total FMS, object control skills, and product-oriented assessments, in comparison to females (*p* < 0.05). Children of Black and White ethnic groups achieved significantly greater mastery of locomotor skills, compared to Asian children, though this did not differ by year-group (*p* < 0.05). The results suggest that FMS development in deprived and ethnically diverse areas in England varies between genders during middle-childhood and ethnicity. Thus, interventions addressing the lack of FMS mastery achievement, shown in middle-childhood girls and children from Asian ethnic backgrounds, may be pivotal. Further exploration of the role of ethnicity would provide greater clarity in approaching interventions to improve FMS.

## 1. Introduction

Regular physical activity (PA) in childhood is essential for lifelong physiological, psychological, cognitive, and social development [1]. However, children from as early as five years old in England demonstrate inactive lifestyles [2], and this inactive behavior increases with age [2,3,4]. Engagement in PA during adolescence and into adulthood is, in part, impacted by a child’s mastery level of Fundamental Motor Skills (FMS) [5]; where high FMS ability promotes a more physically active lifestyle and a low FMS ability promotes a more sedentary lifestyle [5]. The acquisition of FMS initiates during infancy and is commonly acquired by the age of five to six years [6,7]. Children then enter into a phase of transition (seven and ten years), where acquired skills are expected to be mastered and become more context specific—such as in sporting situations [6]. FMS acquisition does not develop naturally or automatically, it must be taught based on age and stage of learning, and should be practiced and reinforced [8,9,10].

The development of FMS has been reported to differ between children from different countries [11], although ethnicity is rarely reported/considered in the analysis [11,12,13,14,15]. Newell’s constraints theory [16] highlights the influence of individual, environmental, and task constraints on motor development. A number of different constrains, such as deprivation, PA engagement levels, and obesity have been shown to relate to ethnicity and FMS exclusively, but have rarely been considered together [17,18,19,20]. For example, research suggests that children in low-socioeconomic (SES) areas perform FMS at a significantly lower level than those in high-SES areas in England [13]. Within England, there is a greater likelihood that ethnic minority groups will live in deprived neighborhoods [21]. Understanding the differences in FMS mastery of children from different ethnic backgrounds, and the constraints relating to it, may further complete the research-base; providing greater insight into FMS development and subsequently PA engagement and future health, particularly within low-SES communities.

The current study seeks to address key gaps in the FMS literature relating to age, gender, and ethnicity while considering deprivation and the controlling of maturation and BMI; examining differences in FMS mastery achievement of early-childhood (EC; four to five years) and middle-childhood (MC; nine-to-ten years) males and females from different ethnic groups.

## 2. Materials and Methods

### 2.1. Sample Selection

Following institutional ethics approval, a cross-sectional study design was employed. Cluster sampling was used to recruit participants in EC (four-to-five years) and MC (nine-to-ten years) from schools in low-SES areas in England. SES was identified by The Local Concentration Measure [21], which is used to rank local authorities in ‘relation to deprivation levels experienced by the most deprived 10% of the local population’ [21]. Concentrating the recruitment in low-SES areas and comparing schools within these areas aimed to control deprivation. Informed consent from parents and the assent of the children was attained. For inclusion in the study, children had to be ‘apparently healthy’ as indicated by parents, from reception, or by year five pupils, and attend a school in England located in the wards, which is classified among the most deprived 20% in England [22,23]. Participants were excluded if the child was unable to take part post-consent (e.g., broken limb, illness, special educational/motor development needs) or if they did not wish to participate (*n* = 5). In total, 293 children in EC (153 males; 4.92 ± 0.27 years) and 161 children in MC (86 males; 9.56 ± 0.5 years) were initially assessed. A prior power calculation indicated that, assuming unequal groups, to detect a medium effect at *p* = 0.05 at 95% power a sample size of 236 was needed.

### 2.2. Anthropometric Data

Height (cm) and seated height (cm) were measured using a stadiometer (Seca 213, Hamburg, Germany). An anthropometric tape measure (HaB direct, Southam, UK) was used to measure waist circumference (cm) and leg length (cm). Waist circumference was taken from the mid-point between the tenth rib and the iliac crest—measured to the nearest mm. Leg length was measured from the greater trochanter to the lateral malleolus. Body mass (kg) was measured using Tanita scales (Tanita BF305, Tanita Inc., Tokyo, Japan), which are valid and reliable for use in the pediatric population [24,25]. Participants wore shorts and a T-shirt for anthropometric data collection. Age (birth year and month), gender, and ethnicity were provided through parent self-reports. The ethnicity of participants was classified within the three main categories outlined by the Department of Education (Black, White, and Asian) [26].

### 2.3. Process-Oriented Assessment of Fundamental Motor Skills

FMS proficiency was assessed using the process-oriented measure Test of Gross Motor Development-2 (TGMD-2) protocol as described by Ulrich [27]. Methods of process-oriented measures focus on the quality of the movement pattern demonstrated [28,29].

The testing of FMS was conducted in one class PE lesson of up to 30 children (45–60 min in duration) in the participant’s school hall. Each class was separated into three groups of 10 or less. Three stations: (1) Locomotor skills (run, gallop, hop, leap, and horizontal jump); (2) object control skills (roll, underarm throw, catch, bounce, and two-handed strike); and (3) anthropometric measures were rotated through during the assessment sessions.

The test trials for all skills were video-recorded (Casio video camera, EX-F1, Tokyo, Japan) and analyzed through Quintic Biomechanics v24 (Quintic Consultancy Ltd., Sutton Coldfield, UK) using the TGMD-2 scoring criteria by Ulrich [27]. Two trained researchers conducted the analysis by following two three-hour FMS-video assessment sessions (rating them against the previously rated ‘gold standard’). When assessor agreement was >80% and differed by ≤1 unit for two test trials, training was classed as complete [30]. Intra-class correlation = 0.99, 95% CI 0.98–0.99.

The raw score for each skill component was summed and used to calculate the following:

(a) Subtest raw score; a value indicating the participant’s performance within a subtest (locomotor or object control). Both locomotor (run, gallop, hop, leap, and horizontal jump) and object control (roll, underarm throw, catch, bounce, and two handed strike) subtest scores ranged from 0 to 40.
(b) Total raw score; a composite score was calculated by adding the locomotor and object control subtests, ranging from 0 to 80.

Norm values of the TGMD-2 were formulated using an American sample and previous literature has suggested that cultural differences are important in the proficiency of motor skills [11]. Thus, to reduce the risk of misclassification in the UK sample—used in the current study—raw scores were used.

### 2.4. Product-Oriented Assessment of Fundamental Motor Skills

Product-oriented measures of sprint speed and standing long jump distance were assessed—two measures that are valid and reliable within the child population [31]. Methods of product-oriented measures are concerned with quantitative outcomes produced by movement patterns [28]. Smart Speed Pro light gates (Fusion Sport, Coopers Plains, Australia) were used to measure 10 m sprint speed from a stationary start. Jump distance was measured from the take-off line to the closest landing position of the heel using a long tape measure. Each assessment followed the process-oriented protocol previously described. The fastest sprint time and the longest jump distance, of three attempts, were used in the data analysis. Z-scores (raw score mean − group mean/standard deviation) were calculated for all the measurements. Only sprint speed Z-scores were reversed in order to account for lower raw scores representing faster/better performance [32]. A composite product score for lower limb power was then calculated (sprint Z-score + jump Z-score) [32].

### 2.5. Statistical Analysis

Participant data were only included for analysis if all personal information (age, birth month, ethnicity, gender), anthropometric measures, and two trials for each FMS were recorded. The final sample (Table 1), included within the analysis, consisted of 170 (58%) children in EC (87 males; 4.92 ± 0.26 years, White 66, Asian 73, Black 31) and 109 (67%) children in MC (60 males; 9.6 ± 0.49 years, White 34, Asian 45, Black 50). A prior power calculation indicated that, assuming that there were unequal groups, to detect a medium effect at *p* = 0.05 at 95% power, a sample size of 236 was needed. Children were not included in the analysis if they were absent at testing (78), if ethnicity data were not being provided (38), if process and product scores were missing (54), and/or if there was a withdrawal (5). Body mass index (BMI) was calculated (kg/m^2^). Means and SDs were calculated for all participants for age (years), body mass (kg), height (cm), and BMI (kg/m^2^). Maturation was accounted for in a subsequent analysis; years from and age at maturation was predicted using anthropometric variables, as explained by Mirwald et al. [33], due to previous observations showing the influence of maturation on FMS mastery ability in children [34]. IBM SPSS Statistics (version 24) was used to examine process-oriented FMS mastery (total, object control, and locomotor skills) and product-oriented FMS performance ability of children (males and females) in EC and MC. A two (EC vs. MC) by two (males vs. females) by three (White vs. Asian vs. Black) ways between groups multivariate analysis of covariance (MANCOVA) was conducted. Assumptions regarding normality and homogeneity of variance were not violated. Effect size was calculated using Cohens-*d* (d) as described in Reference [35]. BMI and maturation were used as covariates but were not significant covariates of total FMS (*p* = 0.75 and *p* = 0.77), object control (*p* = 0.32 and *p* = 0.42), locomotor skills (*p* = 0.45 and *p* = 0.72), proficiency or composite product score (*p* = 0.20 and *p* = 0.26) in the population of the current study. Additionally, the BMI was not significantly different between children within the same year groups. Therefore, maturation and BMI were removed from the analysis and a subsequent multivariate analysis of variance (MANOVA) was carried out. A Bonferroni post-hoc analysis was carried out to identify where any significant differences existed. A Pearson correlation analysis was also conducted between composite product Z-scores and the total FMS, object control and locomotor skills raw score, respectively.

## 3. Results

**Figure 1:**
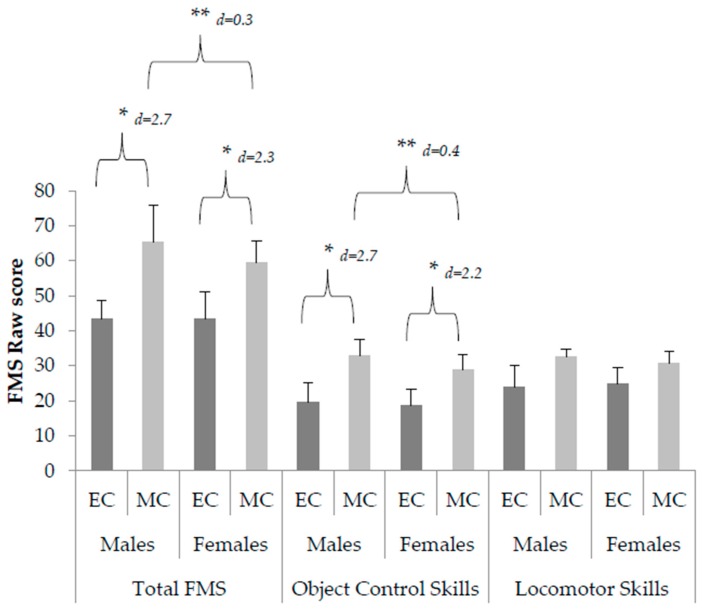
Total Fundamental Motor Skills and object control skill proficiency scores (mean ± standard deviation) of males and females in early and middle-childhood. * Indicates significant difference between age groups (*p* < 0.05). ** Indicates significant difference between males and females in middle-childhood (*p* < 0.05).

The results of the MANOVA, for process and product-oriented FMS, revealed a significant gender (male vs. female) by childhood year (EC vs. MC) interaction (Figure 1). A post-hoc pairwise comparison (Bonferroni adjusted) identified the total FMS mastery of males and females in MC to be significantly better than EC males and females (F_(1,267)_ = 7.27, *p* = 0.007; M1 − M2 = 22.02, 95% CI (21.12, 21.81) and M1 − M2 = 16.20, 95% CI (15.47, 16.47), Figure 1). MC males and females also performed object control skills significantly better than EC males and females (F_(1,267)_ = 5.99, *p* = 0.02; M1 − M2 = 13.39, 95% CI (13.01, 13.39) and M1 − M2 = 10.21, 95% CI (9.76, 10.22), Figure 1).

**Figure 2:**
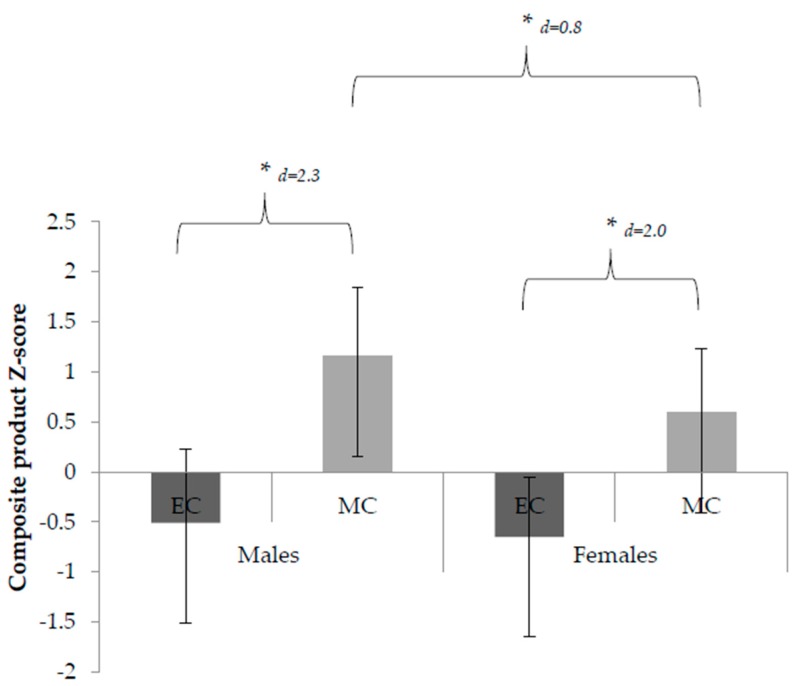
Composite product Z-score (mean ± standard deviation) of early-childhood (EC) and middle-childhood (MC) males and females. * Indicates significant difference between early-childhood and middle-childhood (*p* < 0.05); * Indicates significant difference between males and females within a year group (*p* < 0.05).

Additionally, composite product scores also showed a gender by childhood year interaction (F_(1,267)_ = 5.95, *p* = 0.02), where males and females in MC outperformed those in EC (M1 − M2 = 0.64, 95% CI (0.36, 0.99) and M1 − M2 = −0.06, 95% CI (−0.36, 0.39), Figure 2.

There was a significant childhood year (EC vs. MC) by gender (males vs. female) interaction for both process mastery and the product-oriented performance of children in MC, but not for those in EC. Gender differences were shown in the total FMS mastery of males and females in MC (M1 − M2 = 5.87, 95% CI (6.43, 5.34), *r =* 0.93); *p* < 0.05, Figure 1). Males also demonstrated significantly better object control skills (M1 − M2 = 4.27, 95% CI (4.59, 4.09), *p* < 0.05, Figure 1) and composite product score (M1 − M2 = 0.56, 95% CI (0.58, 0.51), *p* = 0.02, Figure 2).

Overall, children in MC significantly outperformed children in EC in total FMS (M1 − M2 = 19.4, 95% CI (18.43, 18.91), *p* < 0.01), object control raw score (M1 − M2 = 12.00, 95% CI (11.45, 11.75), *p* < 0.01), locomotor raw score (M1 − M2 = 7.4, 95% CI (6.94, 7.22), *p* < 0.01), and composite product score (M1 − M2 = 0.32, 95% CI (0.10, 0.59), *p* < 0.01). As shown in Table 2 and Table 3, a greater percentage of children in MC achieved all components across locomotor and object control skills. The run, leap, and gallop are shown to have had the greatest percentage achievement of all locomotor skills components (67.89%, 56.88%, and 38.5%, respectively), while object control skills were the highest in catch (85.32%), strike (50.46%), and overarm throw (37.61%). However, no skill was mastered by the entire MC sample.

**Figure 3:**
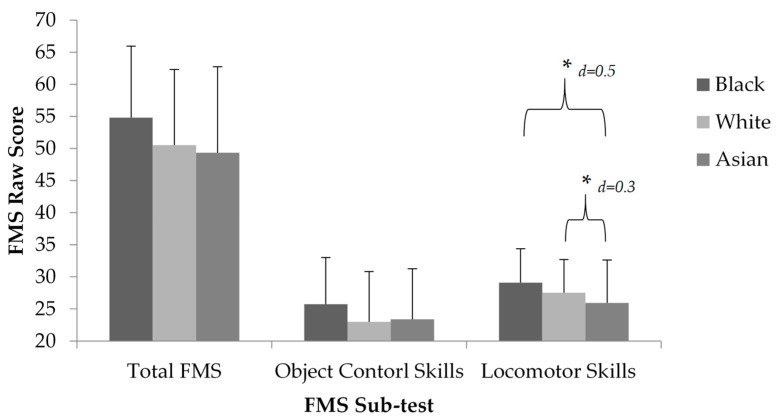
FMS proficiency scores (mean ± standard deviation) by ethnic group (Black vs. White vs. Asian) of total FMs, object control skills, and locomotor skills. * Indicates significant difference between ethnic group *p* < 0.05.

**Figure 4:**
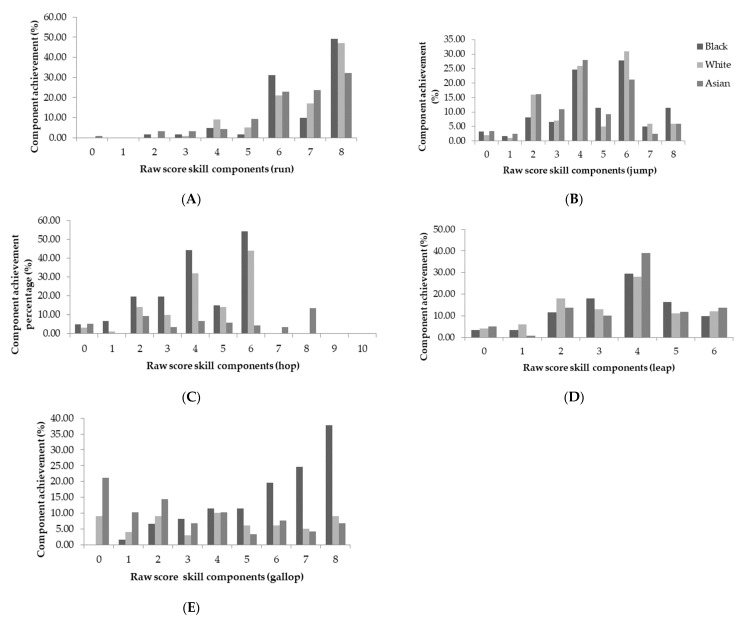
Component achievement (%) of locomotor skills by ethnicity; (**A**) Run; (**B**) Jump; (**C**) Hop; (**D**) Leap; and (**E**) Gallop.

**Figure 5:**
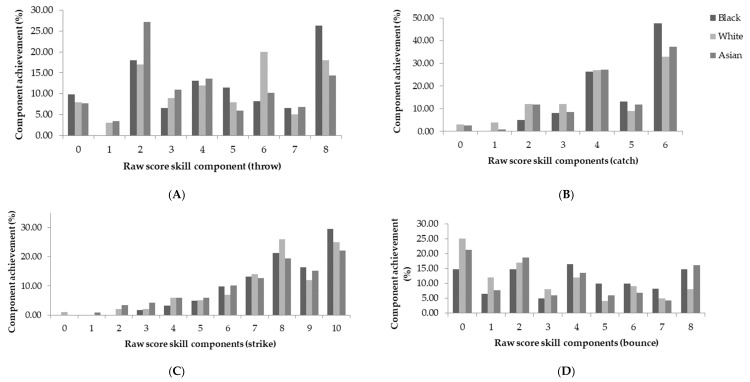
Component achievement (%) of object control skills by ethnicity; (**A**) Throw; (**B**) Catch; (**C**) Strike; and (**D**) Bounce.

When the sample was categorised by ethnicity, a main effect was indicated for locomotor skills (*p* = 0.012; Figure 3). A Bonferroni adjusted post-hoc pairwise comparison indicated similar mastery by Black and White children (Black vs. White; M1 − M2 = 1.55, 95% CI (0.19, 0.72)). Children of both Black and White ethnic groups demonstrated better locomotor skills, compared to Asian children (Black vs. Asian; M1 − M2 = 3.13, 95% CI (1.66, 2.41), White vs. Asian; M1 − M2 = 1.58, 95% CI (1.47, 1.69)). Considering the components of locomotor skills, achieved by participants (Figure 4), children of a Black and Asian ethnic background achieved the greatest percentage of mastery in the leap (55.47%, 37.29%). White children had the greatest achievement of components in the run (47%). Additionally, Black, Asian, and White children all had the greatest component achievement (mastery) in the catch (55.74%, 39.83%, and 39%). Across all ethnicities, no skill was mastered, and the hop and the roll were the least mastered skills by all participants (Figure 4 and Figure 5).

## 4. Discussion

This study sought to identify differences in the process and product FMS ability of children in early and middle-childhood who attend schools in deprived and ethnically diverse areas in England—while considering gender and ethnic background. Novel data are presented through assessing children in the acquisition phase in EC and at the end of the transitional stage in MC of FMS development in England, comparing year group, gender, and ethnicities between and within these groups. The key finding from this study is that children from Black and White ethnic backgrounds performed significantly better for locomotor skills, compared to Asian children, irrespective of childhood stage. Eyre et al.’s [32] work is the only study to have observed the role of ethnicity and FMS in five-year-olds, reporting that South Asian children demonstrated poorer total FMS and locomotor skills, compared to Black and White children. The current study extends the work of Eyre et al. [32], showing that the ethnic differences in locomotor skills reported by Eyre et al. [32] also persist in middle-childhood. Across developmental time, FMS have been suggested to be impacted by a combination of multiple interlinking constrains relating to the individual, their environment, and the task at hand [16]. These may include exposure to, and experience of, PA engagement; children from South Asian backgrounds living in England have the greatest percentage of children failing to meet the UK PA recommended guidelines for health, compared to their Black and White counterparts [36,37,38]. Thus, Asian children may experience less opportunity for FMS to be practiced and reinforced [8,9,10]. Secondly, social interactions and the influence of cultural norms/expectations, encouragement from significant others/role models, and/or what their family/friends engage in within different ethnic communities. Constraints may also be found in the community environment (e.g., socio-economic status), the lack of safe open spaces, and the equipment available for engagement/practice—as well as finances available for equipment and clubs/projects engagement [39,40]. Thus, FMS development relating to ethnicity may be multifaceted, with Asian children undertaking greater constraints during childhood than Black and White children resulting in the lack of mastery achievement seen in the current study. The current findings develop the limited research in the UK surrounding ethnicity and deprivation, though it is clear that further investigation is necessary.

While ethnic differences did not differ by age, the current study also showed a greater mastery of skills from children in MC compared to those in EC—complementing the literature [20]. A main founding pillar of FMS development is the need for teaching, practice, and the reinforcement of skills [9]. Therefore, within the current study, as older children are expected to be in the latter transitional stages of the PE curriculum in England, having greater engagement by being taught and practicing skills may have, in part, facilitated FMS development [41]. If PE is engaged in there may be greater potential for strengthening the synaptic pathways and motor control strategies and the experience of synaptic pruning over a greater period of time in MC, compared to children in EC [34]. It should still be considered that the rate of each child’s progression through the developmental phases and stages are individual, and that development is related to age but not necessarily dependent upon it [6]. When comparing mastery achievement of participants in the current study to the TGMD-2 standardization sample [27], limited achievement is proposed in both age groups—particularly the children in MC as they are all expected to have mastered FMS. The only skills to show a greater percentage mastery achievement were the leap during EC and MC (+20.12% and +6.88%), as well as the strike and catch during MC (+1.46% and +2.32%). Mastery of all other skills in the current study was below the percentage achieved by the standardized sample [27]. This comparison should be considered with caution, considering differences in FMS mastery across different countries and as the standardisation sample was based in America [11,27]. It is clear that age is not the sole determinant of FMS mastery.

Gender differences were also highlighted in the current study, with MC males performing better than females in total FMS and object control skills, aligning with much of the literature [13,42]. This has previously been attributed to males being more physically active than females, and being encouraged during EC to engage in ‘masculine’ activities (e.g., football) [7]. Furthermore, males have been found to engage in PA with a greater use of ball control elements, compared to the rhythmic and balance elements engaged in by females [43]. Considering this and the findings that show childhood playmate selection is often based on gender [44], contributing factors to the differences in mastery attainment between genders vary from individual to individual as well as social and environmental constructs. Interestingly however, locomotor skills of males and females were not significantly different in the current study. This finding, however, contradicts the literature showing a greater mastery of locomotor skills in females [20], where a similar sample (multi-ethnic children from low socio-economic areas in the UK) and methods were used by Eyre et al. [32] who also found no gender differences in locomotor skills of children in EC. Gender similarities propose that males and females in deprived and ethnically diverse areas may undergo fewer differences in regards to constraints relating to locomotor skills and where the rate of FMS development may be comparable. It should also be noted that studies that have found differences in locomotor skills differ in FMS assessment tools, populations (age, ethnicity), geographical location/environment, and subsequently societal norms/expectations as well as the SES from the current study. Thus, the present findings broaden the understanding of FMS mastery attainment of children in England and its relation to ethnicity and deprivation.

As in much of the literature, the current study presents its own limitations and strengths. Firstly, the cross-sectional nature of the study means that the level and rate of FMS developmental progression from EC to MC could not be observed. Future research should aim to conduct longitudinal research to provide a broader picture of FMS development across developmental time, which will subsequently inform FMS interventions in relation to gender specific time points as well as possible ethnic patterns/trends if any. Additionally, deprivation was accounted for in relation to the school attended by participants. Further, specification of post-codes lived in by participants along with the comparison between ethnically diverse schools in high and low-SES areas may provide a greater scope and understanding of varying FMS mastery achievement by SES and the ethnicities within them. The current study does, however, develop the sparse research base relating to ethnicity and FMS; when deprivation, maturation, and obesity are controlled for lack of mastery achievement, it is present in Black, White, and Asian children, overall. Moreover, Black and White children in EC and MC seem to be more proficient in FMS than those from Asian ethnic backgrounds. Future research would benefit from further investigation of the FMS constraints highlighted in the literature [45], and in relation to ethnicity, the categorizing of sub-groups within ethnicities. Despite the sample of the current study not being big enough to conduct a sub-group analysis, this line of investigation may further distinguish findings relating to FMS within ethnic groups. Highlighting these may help inform interventions targeting groups that display a reduced mastery achievement that were expected to reduce the proficiency gap and subsequently improve the health trajectories of children.

## Figures and Tables

**Table 1:** Participant demographic variables by year group and gender.

	All (Mean ± SD)	Early Childhood (Mean ± SD)		Middle Childhood (Mean ± SD)		Ethnicity (Mean ± SD)		
		Males	Females	Males	Females	Black	White	Asian
*n*	279	87	83	60	49	61	100	118
Age (years)	6.76 ± 2.32	4.93 ± 0.26	4.93 ± 0.26	9.60 ± 0.49	9.60 ± 0.49	7 ± 2.30	7 ± 2.32	7 ± 2.31
Standing Height (cm)	122.20 ± 15.96	111.10 ± 6.29	111.10 ± 6.33	139.71 ± 9.83	139.52 ± 9.96	122.10 ± 16.01	122.3 ± 16.01	122.2 ± 16.01
Seated Height (cm)	62.64 ± 6.57	58.09 ± 3.11	58.09 ± 3.13	69.82 ± 3.50	69.73 ± 3.54	62.60 ± 6.58	62.7 ± 6.59	62.6 ± 6.59
Mass (kg)	26.51 ± 10.27	19.89 ± 3.61	19.91 ± 3.62	37.06 ± 8.56	36.83 ± 8.75	26.48 ± 10.30	26.6 ± 10.30	26.5 ± 10.30
BMI (kg/m^2^)	17.18 ± 3.61	16.03 ± 1.84	16.04 ± 1.84	19.05 ± 4.79	18.98 ± 4.83	17.18 ± 3.61	17.2 ± 3.61	17.2 ± 3.62
Waist Circumference (cm)	53.51 ± 11.06	52.02 ± 3.95	52.01 ± 3.96	56.03 ± 16.94	55.75 ± 16.97	53.7 ± 12.18	52.42 ± 10.95	54.3 ±10.59

**Table 2:** Component achievement (%) of locomotor skills during early-childhood (EC) and middle-childhood (MC).

Components Achieved	Run (%)		Gallop (%)		Hop (%)		Leap (%)		Jump (%)	
	EC	MC	EC	MC	EC	MC	EC	MC	EC	MC
0	0.59	0.00	4.71	0.00	7.06	0.00	2.35	0.00	4.12	0.92
1	0.00	0.00	1.18	0.00	1.76	0.00	2.94	0.00	2.35	0.92
2	2.94	0.00	8.82	5.50	4.71	0.00	15.88	1.83	21.76	2.75
3	3.53	0.00	8.82	0.92	5.88	0.92	7.65	3.67	13.53	0.92
4	9.41	0.92	19.41	2.75	17.65	3.67	24.12	18.35	32.35	17.43
5	8.82	1.83	8.24	7.34	8.82	6.42	12.94	19.27	8.82	7.34
6	30.00	5.50	22.94	33.03	25.29	33.03	34.12	56.88	12.35	47.71
7	20.59	14.68	12.35	11.93	11.76	21.10			1.76	9.17
8	24.12	67.89	13.53	38.53	17.06	34.86			2.94	13.76
9					0.00	0.00				
10					0.00	0.00				

**Table 3:** Component achievement (%) of locomotor skills during early-childhood (EC) and middle-childhood (MC).

Components Achieved	Strike (%)		Bounce (%)		Catch (%)		Overarm Throw (%)		Roll (%)	
	EC	MC	EC	MC	EC	MC	EC	MC	EC	MC
0	0.59	0.00	32.35	3.67	3.53	0.00	12.35	1.83	5.88	1.83
1	0.59	0.00	13.53	1.83	2.94	0.00	4.12	0.00	4.12	1.83
2	2.94	0.92	25.88	3.67	17.06	0.00	27.06	12.84	18.82	8.26
3	4.71	0.00	7.06	5.50	15.88	0.00	12.94	3.67	14.12	11.01
4	8.24	0.92	12.35	15.60	37.06	11.01	17.06	6.42	37.65	25.69
5	7.65	1.83	4.71	8.26	15.88	3.67	8.24	7.34	12.94	11.93
6	10.59	6.42	2.35	17.43	7.65	85.32	10.59	17.43	4.12	24.77
7	19.41	3.67	0.59	11.93			1.76	12.84	1.18	4.59
8	25.29	17.43	1.18	32.11			5.88	37.61	1.18	10.09
9	11.18	18.35								
10	8.82	50.46								
